# Heparin-Binding Hemagglutinin-Induced Trained Immunity in Macrophages: Implications for Antimycobacterial Defense

**DOI:** 10.3390/biom15070959

**Published:** 2025-07-04

**Authors:** Yongqiang Li, Xiuping Jia, Jinhua Tang, Huilian Qiao, Jiani Zhou, Yueyun Ma

**Affiliations:** 1 Department of Clinical Laboratory, Air Force Medical Center, Beijing 100142, China; lyq921004@163.com (Y.L.); tang4113@163.com (J.T.); qiaohi330@fmmu.edu.cn (H.Q.); 2 College of Life Science, Northwest University, Xi’an 710069, China; 202233085@stumail.nwu.edu.cn (X.J.); 202434394@stumail.nwu.edu.cn (J.Z.)

**Keywords:** heparin-binding hemagglutinin, trained immunity, macrophage, tuberculosis, epigenetic reprogram

## Abstract

Tuberculosis (TB) is a major global health threat, with the current *Bacillus Calmette–Guérin* (BCG) vaccine having limited efficacy against adult pulmonary disease. Trained immunity (TI) is a form of innate immune memory that enhances antimicrobial defense. It is characterized by the epigenetic and metabolic reprogramming of innate immune cells and holds promise as a promising approach to prevent TB. In this study, we investigated the capacity of heparin-binding hemagglutinin (HBHA), a methylated antigen of *Mycobacterium tuberculosis*, to induce TI in murine RAW264.7 macrophages, human-derived THP-1 macrophages, and human peripheral blood mononuclear cells (hPBMCs). HBHA-trained macrophages exhibited the enhanced expression of pro-inflammatory cytokines (IL-1β, IL-6, TNF-α) following secondary lipopolysaccharide stimulation. The epigenetic profiling indicated elevated levels of H3K4me1 and H3K4me3 histone marks at cytokine gene loci. Further, metabolic analysis revealed heightened lactate production and the increased expression of glycolytic enzymes. Functionally, HBHA-trained macrophages exhibited improved control of intracellular mycobacteria, as evidenced by a significant reduction in colony-forming units following BCG infection. These findings elucidate that HBHA induces a functional TI phenotype via coordinated epigenetic and metabolic changes, and suggest HBHA may serve as a valuable tool for studying TI and its relevance to host defense against mycobacterial infections, pending further in vivo and clinical validation.

## 1. Introduction

Tuberculosis (TB), caused by *Mycobacterium tuberculosis* (Mtb), is a significant global health challenge, with 8.2 million new cases and 1.25 million deaths reported in the World Health Organization’s Global Tuberculosis Report 2024 [1]. *Bacillus Calmette–Guérin* (BCG), the only licensed TB vaccine, provides partial protection against disseminated TB in children but shows variable efficacy (10–50%) against pulmonary TB in adults [2,3], highlighting the urgent need for novel vaccination strategies.

Recent advances in immunology have identified trained immunity (TI)—the epigenetic and metabolic reprogramming of innate immune cells—as a promising approach to bolster antimicrobial defense [4,5,6]. Unlike classical adaptive immunity, TI enables innate cells, such as macrophages and monocytes, to mount heightened responses to subsequent infections, including those caused by unrelated microbes [4,7]. Although systemic TI induction by BCG or β-glucan in circulating monocytes is well-characterized [8,9], TI has recently been observed in tissue-resident innate immune cells, including alveolar macrophages, following respiratory mucosal exposure [10,11,12]. This localized TI in the lung holds particular promise for pulmonary pathogens like Mtb, where alveolar macrophages serve as the first line of defense and are critical in orchestrating early immune responses [13,14].

However, the innate immune activation of alveolar macrophages is subverted by Mtb through evolved mechanisms, including resistance to both reactive oxygen and nitrogen intermediates and the inhibition of phagosome–lysosome fusion. This immune evasion delays the migration of antigen-presenting cells (APCs) and priming of Th1, rendering the lung vulnerable for 2–3 weeks post-exposure [15,16]. Traditional parenteral TB vaccines, primarily focused on inducing T cell immunity, are limited by delayed T cell recruitment to lung mucosa [17]. In contrast, the induction of TI in alveolar macrophages could establish a primed antimicrobial state, enabling rapid and robust responses to Mtb, potentially overcoming these early vulnerabilities [15,18]. Although respiratory mucosal vaccination strategies, such as adenovirus-vectored TB vaccines, have been demonstrated to have the capacity to reprogram alveolar macrophages for TI [15,19], challenges such as safety concerns and pre-existing immunity highlight the need for alternative candidates to be developed.

Heparin-binding hemagglutinin (HBHA), a 28 kDa methylated protein encoded by Mtb *Rv0475* [20], has emerged as a novel candidate for inducing TI. Beyond facilitating bacterial aggregation and dissemination, native HBHA’s C-terminus, which contains methylated lysine-repeat sequences, confers potent immunogenicity, [21,22]. Our laboratory has successfully produced recombinant methylated HBHA [23], which is known to stimulate strong T cell responses and confer protective immunity in murine models of tuberculosis [24,25,26]. Notably, HBHA is also a toll-like receptor 4 (TLR4) agonist that possess a strong immunostimulatory potential; it can induce DC maturation in a TLR4-dependent manner [27,28]. However, its capacity to epigenetically and metabolically reprogram macrophages for TI remains unexplored, particularly in the context of TB.

In this study, we investigate the capacity of HBHA to induce TI in the RAW264.7 macrophage cell line, which is a well-established in vitro model for innate immune responses. Consequently, HBHA training enhanced the key hallmarks of TI, including the production of pro-inflammatory cytokines, enrichment of histone methylation marks at cytokine gene loci, and metabolic alterations in glycolysis-related pathways. Functionally, HBHA-trained macrophages exhibited improved control of intracellular mycobacteria, evidenced by reduced colony-forming units (CFUs) post-BCG infection. Moreover, the TI phenotype was further validated in human THP-1-derived macrophages and peripheral blood mononuclear cells (hPBMCs), underscoring its translational potential. Together, our results elucidate the molecular basis of HBHA-induced TI and highlight HBHA could be a promising candidate for further investigation in the context of tuberculosis prevention.

## 2. Materials and Methods

### 2.1. Reagents and Antibodies

The recombinant HBHA-expressing *Mycobacterium smegmatis* (MS) strain mc^2^155(rHBHA-MS) was constructed prior to this study and maintained in our laboratory. Recombinant HBHA protein was produced and purified following an established protocol [23]. RPMI1640 medium, fetal bovine serum (FBS), and penicillin–streptomycin liquid were purchased from Gibco (Waltham, MA, USA). Chromatin immunoprecipitation (ChIP)-grade primary antibodies against H3K4me1 and H3K4me3 and rabbit IgG control antibody were purchased from Abcam (Cambridge, MA, USA). An ELISA kit for the detection of mouse IL-1β, IL-6, and TNF-α was purchased from Dakewe (Shenzhen, China). A lactate assay kit was purchased from Solarbio (Beijing, China). Finally, the ChIP assay kit was purchased from Beyotime (Shanghai, China).

### 2.2. Cell Lines and Cell Isolation

The murine macrophage cell line RAW264.7 and human monocyte cell line THP-1 were obtained from Procell Life Science & Technology Co., Ltd. (Wuhan, China). Fresh hPBMCs were isolated venous blood of healthy donors, which had been treated with the ethylenediaminetetraacetic acid (EDTA) as an anticoagulant. The isolation was carried out using gradient centrifugation using Ficoll-Paque. All cells were cultured in RPMI1640 medium containing 10% fetal bovine serum, 100 units/mL of penicillin, and 100 μg/mL of streptomycin. The cells were incubated in a humidified incubator at 37 °C with 5% CO_2_. This study approved by the Ethics Committee of the Air Force Medical Center, PLA (Protocol #2025-61-S01, 2025-01-08).

### 2.3. Trained Immunity in Macrophages and hPBMCs

RAW264.7, THP-1, or hPBMCs were seeded in 6-well flat-bottom plates. On day 0, the cells were incubated with HBHA in their indicated concentrations (1 μg/mL or 6 μg/mL) for 24 h at 37 °C. The cells were then washed three times with PBS and cultured in RPMI1640 for 5 days rest, and the 5-day rest period was selected based on prior TI models [29,30]. On day 6, the cells were rewashed and subsequently restimulated with 10 ng/mL of lipopolysaccharide (LPS) derived from *Escherichia coli* O111:B4 (Beyotime, Shanghai, China). After 24 h, the cells were collected for total RNA extraction, and supernatants were harvested and preserved at −80 °C for subsequent cytokine analysis.

### 2.4. Quantitative Real-Time PCR

Cells with or without LPS restimulation, as described above, were collected, and their total cellular RNA was extracted using the FastPure Cell/Tissue Total RNA Isolation Kit V2 (Vazyme, Nanjing, China). The extracted RNA was then reverse-transcribed into cDNA using the Primescript RT Master Kit (Takara, Kyoto, Japan), following the manufacturer’s instructions. Quantitative real-time PCR was subsequently performed using the SYBR Premix Ex Taq Kit (Takara, Kyoto, Japan) on Bio-Rad CFX96 Real-time System (Bio-Rad, Hercules, CA, USA). The PCR amplification protocol included an initial denaturation at 94 °C for 30 s, followed by 40 cycles of 94 °C for 25 s, 60 °C for 45 s, and 72 °C for 45 s. The specific primers targeting IL-1β, IL-6, TNF-α, and key enzymes in glycolysis (as listed in Table 1) were employed [31]. The Gene expression levels were analyzed using the 2^−ΔΔCt^ method.

### 2.5. Cytokine Assay

Supernatants from LPS-restimulated cell cultures were harvested, and the concentrations of IL-1β, IL-6, and TNF-α in the supernatants were quantified using ELISA kits, respectively, following the protocols provided by the manufacturer. Absorbance values were recorded using a microplate reader set to 450 nm for detection, with 620 nm serving as the reference wavelength.

### 2.6. In Vitro Mtb Killing Assays

RAW264.7 cells were trained with 1 μg/mL of HBHA for 24 h and left to rest for another 5 days, as described above. On day 6, both HBHA-trained and untrained RAW264.7 cells were cultured in RPMI1640 medium containing 10% FBS and subsequently infected with BCG at MOI = 10:1. Four hours post-infection, extracellular BCG was removed by washing, and the cells were lysed using 0.02% SDS for 15 min at 37 °C. The cell lysates were subjected to serial dilution and plated onto Middlebrook 7H10 agar plates to determine bacterial counts at 4, 24, and 48 h post-infection.

### 2.7. Determination of Lactate

RAW264.7 cells were treated with 1 μg/mL of HBHA, as described previously. At the end of the 5-day resting phase, culture supernatants were collected. Lactate concentrations in these supernatants were measured using the colorimetric lactate assay kit (Solarbio, Beijing, China). Absorbance values were measured at 570 nm using a microplate reader.

### 2.8. ChIP Assay

RAW264.7 cells were seeded into 6-well flat-bottom plates. On day 0, the cells were treated with 1 μg/mL of HBHA for at 37 °C for 24 h. After treatment, the cells were rinsed three times with PBS and subsequently maintained in RPMI1640 for 5 days. The ChIP assay was conducted using the ChIP assay kit, adhering to the manufacturer’s instructions. Briefly, the cells were first crosslinked with 1% formaldehyde followed by chromatin fragmention via sonication. Sheared chromatin that containing 100 μg of protein was adjusted to a total volume of 1000 μL. A 20 μL portion was saved for 2% input. The remaining sample underwent preclearing with Protein A + G Agarose/Salmon Sperm DNA, then incubated overnight at 4 °C with 2 μg of CHIP-grade antibodies targeting H3K4me1 or H3K4me3. DNA–protein complexes were captured, and after decrosslinking, DNA was purified using the DNA purification kit (Takara, Kyoto, Japan). The amount of immunoprecipitated DNA was determined by real-time PCR using the SYBR green kit and the Bio-Rad CFX96 Real-time System (Bio-Rad, Hercules, CA, USA), with primers listed in Table 2 [32,33,34]. The PCR products were analyzed to quantify enrichment relative to the input DNA using the formula: input% =2% × 2^ (CT^input^ − CT^sample^) [35].

### 2.9. Statistical Analysis

All values were expressed as mean ± SD. Data were analyzed using Student’s *t*-test for comparisons between two groups, and one-way ANOVA or two-way ANOVA for multiple group comparisons. Post hoc analyses were conducted using the least significant difference (LSD) test to evaluate the differences between groups. Differences with *p*-values <0.05 were considered statistically significant. Outcome blinding was not performed in this exploratory in vitro study, which may introduce potential bias.

## 3. Results

### 3.1. HBHA Training Enhances Production of Pro-Inflammatory Cytokines in RAW264.7

**Figure 1:**
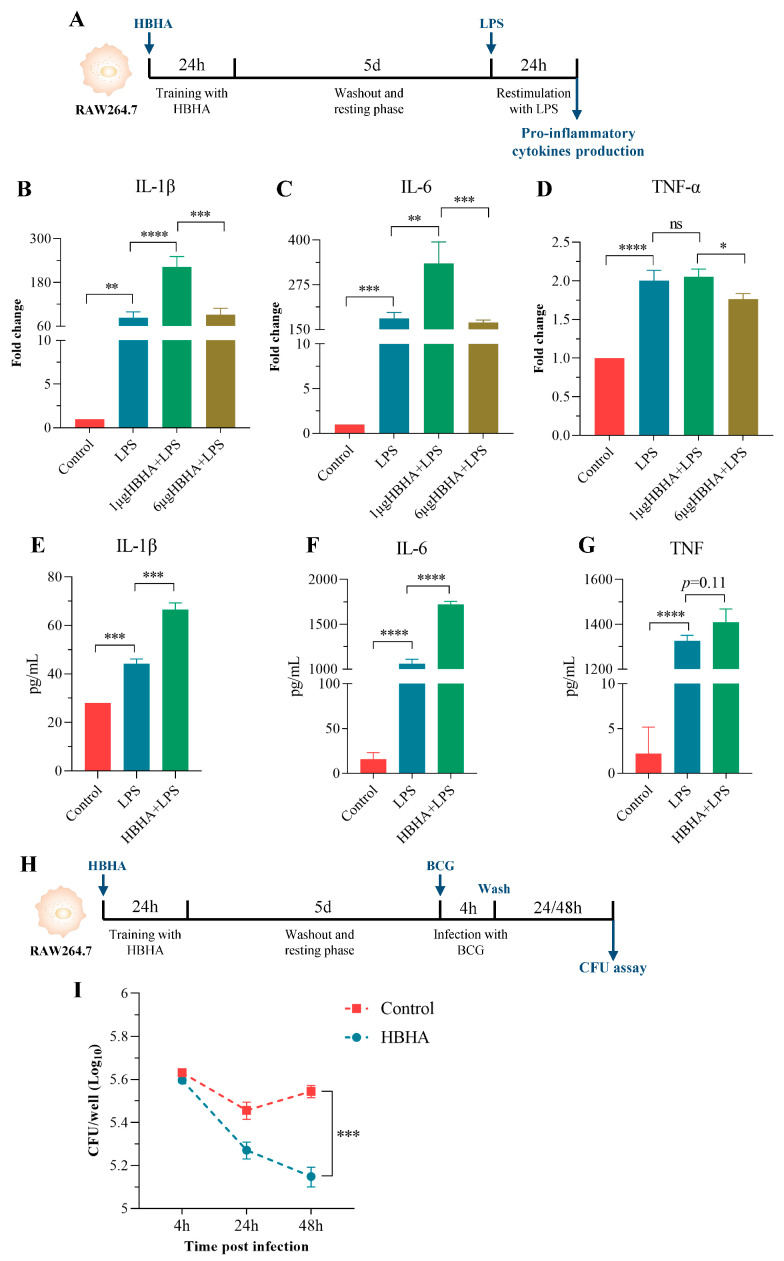
HBHA-trained RAW264.7 exhibits enhanced cytokine production and antimycobacterial activity. (**A**) Experimental schema of HBHA training RAW264.7 in vitro. RAW264.7 macrophages were trained with HBHA (1 or 6 μg/mL) for 24 h, left to rest for 5 days, and restimulated with LPS (10 ng/mL) for 24 h to assess the cytokine responses. The control groups included untreated cells and cells stimulated with LPS alone, without prior HBHA training. (**B**–**D**) The relative mRNA expression of IL-1β, IL-6, and TNF-α in RAW264.7 cells following LPS restimulation was measured by qRT-PCR. The experimental groups include HBHA-trained cells (1 or 6 μg/mL), LPS-only treated cells, and a non-treated control group. (**E**–**G**) The concentrations of IL-1β, IL-6, and TNF-α in the culture supernatants after LPS restimulation were measured using an ELISA. The control groups consist of LPS-only treated cells and non-treated control cells. (**H**) Schematic of the CFU assay. RAW264.7 cells were trained with HBHA, infected with BCG, and bacterial loads were quantified over time. (**I**) Bacterial burden in RAW264.7 macrophages at 4, 24, and 48 h post-infection. CFU counts per well were determined and presented as log10 values. The control group included untrained RAW264.7 macrophages that were infected with BCG. Data in (**B**–**G**,**I**) represent mean ± SD from three independent experiments (n = 3 biological replicates), each with three technical replicates. One-way ANOVA with Tukey’s post-test was used for multiple comparisons of data in (**B**–**G**). Data in (**I**) were analyzed using two-way ANOVA with Sidak’s post-test to compare the bacterial burden between experimental and control groups. * *p* < 0.05, ** *p* < 0.01, *** *p* < 0.005, and **** *p* < 0.001; ns, not significant.

A defining characteristic of TI is the heightened production of pro-inflammatory cytokines following restimulation with unrelated microbial ligands [5]. To determine whether HBHA induces TI in macrophages, RAW264.7 cells were stimulated with HBHA for 24 h, rested for 5 days, and then restimulated with LPS for 24 h (Figure 1A). The gene expression of key pro-inflammatory cytokines was analyzed using qRT-PCR.

As indicated in Figure 1B–D, training with 1 μg/mL of HBHA, but not 6 μg/mL, significantly upregulated IL-1β and IL-6 expression upon LPS restimulation compared to the LPS-only stimulation group and the non-treated control group that received neither HBHA training nor LPS restimulation. Notably, the expression of TNF-α in the HBHA-trained group showed no significant difference from the LPS-only group, but it was substantially elevated relative to the non-treated control group. These data indicate that HBHA-induced TI is concentration-dependent, with 1 μg/mL of HBHA effectively priming RAW264.7 macrophages for an enhanced inflammatory response.

To confirm these findings at the protein level, we measured the concentrations of IL-1β, IL-6, and TNF-α in the supernatants of RAW264.7 cells trained with 1 μg/mL of HBHA and restimulated with LPS. As shown in Figure 1E–G, HBHA training significantly increased the secretion of IL-1β and IL-6, with a moderate increase in TNF-α levels, corroborating the gene expression results.

Together, these findings establish that HBHA induces TI in RAW264.7 macrophages, amplifying their pro-inflammatory cytokine production, and position HBHA as a potent inducer of macrophage TI.

### 3.2. HBHA-Trained Macrophages Exhibit Enhanced Mycobacterial Killing Capacity

To evaluate the antimicrobial effects of HBHA-induced trained immunity, an in vitro Mtb killing assay was performed using RAW264.7 macrophages infected with *Mycobacterium bovis* BCG. Cells were trained with HBHA (1 μg/mL) for 24 h, left to rest for 5 days, and subsequently infected with BCG at an MOI of 10:1. Bacterial burden was assessed at 4, 24, and 48 h post-infection using enumeration of colony-forming units (CFUs) (Figure 1H).

At 4 h post-infection, the mycobacterial burden was comparable between the HBHA-trained and untrained control groups (untrained RAW 264.7 macrophages), indicating equivalent initial infection rates across the conditions. At 24 h post-infection, a significant reduction in bacterial burden was observed in the HBHA-trained group compared to the untrained control group. This difference became more pronounced at 48 h, suggesting enhanced bacterial clearance in the HBHA-trained macrophages (Figure 1G).

These results demonstrate that HBHA-induced trained immunity enhances the antimicrobial effects of RAW264.7 macrophages, effectively suppressing mycobacterial proliferation in vitro, which underscores the potential of HBHA training to bolster innate immune responses against mycobacterial infections.

### 3.3. HBHA Training Triggers Epigenetic Modifications in RAW264.7 Macrophages

**Figure 2:**
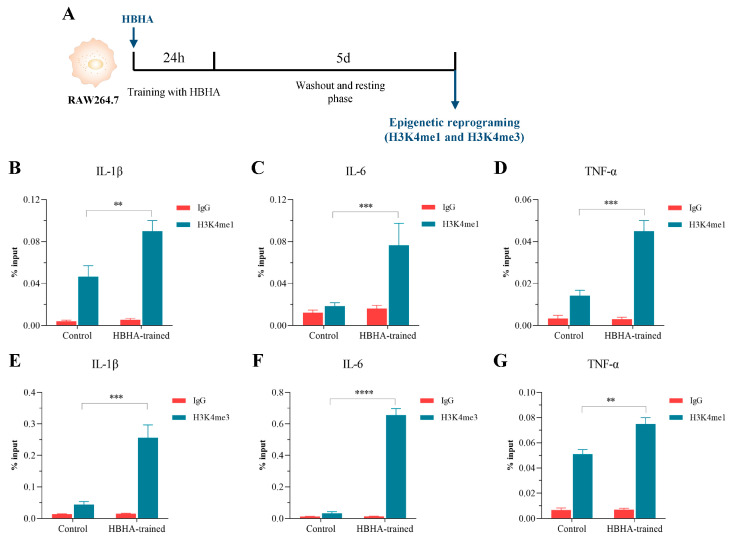
HBHA training induces epigenetic modifications at pro-inflammatory cytokine gene loci in RAW264.7 macrophages. (**A**) Schematic overview of the experimental design. RAW264.7 macrophages were trained with HBHA (1 μg/mL) for 24 h, left to rest for 5 days, and ChIP-qPCR was performed to assess histone modifications at cytokine gene loci. (**B**–**D**) Enrichment of H3K4me1 at the IL-1β, IL-6, and TNF-α gene loci in HBHA-trained and untrained RAW264.7. The control groups consist of untrained control and IgG negative control groups. (**E**–**G**) Enrichment of H3K4me3 at the same loci in HBHA-trained and untrained RAW264.7. The control groups consist of untrained control and IgG negative control groups. Data are presented as a percentage of input chromatin and represent the mean ± SD from three independent experiments (n = 3 biological replicates), each with three technical replicates. Statistical significance for (**B**–**G**) was determined using unpaired Student’s *t*-test. ** *p* < 0.01, *** *p* < 0.005, **** *p* < 0.001.

One of the central mechanisms driving TI is epigenetic modification, which enables an enhanced and accelerated transcriptional response upon subsequent immune challenges [5]. To explore whether HBHA stimulation induces such epigenetic changes in macrophages, RAW264.7 cells were treated with HBHA for 24 h, followed by a 5-day resting period. Subsequently, the cells were harvested for ChIP followed by quantitative PCR analysis (Figure 2A). We examined the enrichment of histone modifications H3K4me1 and H3K4me3 at the promoter or enhancer regions of pro-inflammatory cytokine genes IL-1β, IL-6, and TNF-α using specific antibodies against H3K4me1 and H3K4me3.

As illustrated in Figure 2B–D, ChIP-qPCR analysis with the anti-H3K4me1 antibody demonstrated a significant increase in the percentage of input for IL-1β, IL-6, and TNF-α in the HBHA-trained group compared to both the untrained control and IgG-negative control groups. A parallel trend was observed for H3K4me3 (Figure 2E–G), where the HBHA-trained group showed substantially higher enrichment at these gene loci relative to the untrained control and IgG negative control groups. These findings confirm that HBHA training markedly elevates the levels of histone methylation, specifically H3K4me1 and H3K4me3, at the regulatory regions of IL-1β, IL-6, and TNF-α in RAW264.7 macrophages.

The enhanced deposition of these activating histone marks indicates that HBHA-induced trained immunity in macrophages is associated with epigenetic changes, facilitating the swift and robust expression of IL-1β, IL-6, and TNF-α upon restimulation. This heightened responsiveness underscores the role of HBHA in amplifying the innate immune response through epigenetic reprogramming.

### 3.4. HBHA Training Induces Metabolic Reprogramming in RAW264.7 Macrophages

**Figure 3:**
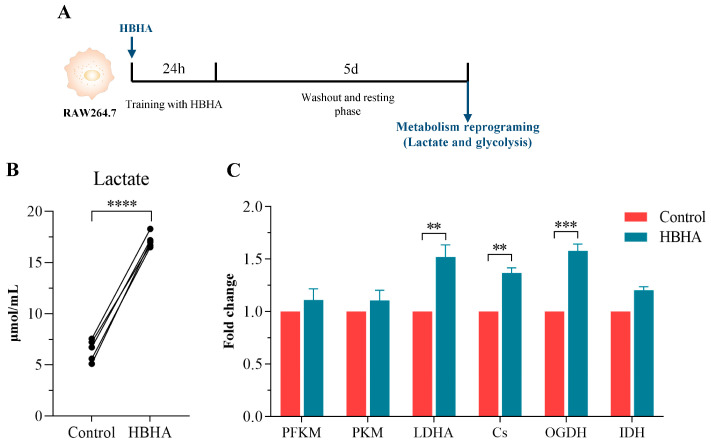
HBHA training induces metabolic reprogramming in RAW264.7 macrophages. (**A**) Schematic overview of the experimental design. RAW264.7 macrophages were trained with HBHA (1 μg/mL) for 24 h, followed by a 5-day resting period before analyzing metabolic reprogramming, including lactate production and glycolysis-related gene expression. (**B**) Lactate concentration in culture supernatants of RAW264.7 cells after HBHA training, compared to the untrained control group. (**C**) Relative mRNA expression levels of key glycolytic and TCA cycle enzymes (PFKM, PKM, LDHA, Cs, OGDH, IDH1) measured via qRT-PCR, compared to the untrained control group. Data are shown as mean ± SD from three independent experiments (n = 3 biological replicates), each with technical replicates. Statistical significance was determined using an unpaired Student’s *t*-test for (**B**) and paired Student’s *t*-test for (**C**). ** *p* < 0.01, *** *p* < 0.005, **** *p* < 0.001.

Metabolic reprogramming toward aerobic glycolysis is a critical feature of TI, providing the biosynthetic intermediates and epigenetic cofactors necessary for immune activation [4]. To determine whether HBHA induces metabolic changes in trained macrophages, we quantified lactate accumulation, which is a surrogate indicator of aerobic glycolysis, and analyzed the gene expression of key enzymes in glycolysis and the tricarboxylic acid (TCA) cycle in RAW264.7 cells that were trained with HBHA for 24 h and left to rest for 5 days (Figure 3A).

As shown in Figure 3B, HBHA-trained cells exhibited a significant increase in lactate accumulation compared to the untrained control group, indicative of enhanced glycolytic activity. Furthermore, qRT-PCR analysis revealed the upregulated transcription of glycolysis-related and TCA cycle enzymes, including phosphofructokinase (PFKM), pyruvate kinase (PKM), lactate dehydrogenase A (LDHA), citrate synthase (Cs), oxoglutarate dehydrogenase (OGDH), and isocitrate dehydrogenase 1 (IDH1). As illustrated in Figure 3C, HBHA training upregulated the expression of all of the examined enzymes relative to the untrained control group. Notably, the increases in LDHA, Cs, and OGDH were statistically significant, highlighting the pivotal roles that they play in enhancing glycolytic flux and TCA cycle activity. The elevated expression of LDHA corroborates the observed increase in lactate production, while the upregulation of CS and OGDH suggests augmented TCA cycle function, potentially fueling the production of epigenetic regulatory intermediates.

These findings demonstrate that HBHA training induces robust metabolic reprogramming in RAW264.7 macrophages, characterized by enhanced glycolysis and TCA cycle activation. This metabolic adaptation provides energy to support immune cell function. In addition, it generates metabolites that synergize with epigenetic modifications to establish a persistent trained state [4,5]. Therefore, these results establish HBHA as a potent inducer of metabolic reprogramming, contributing to the establishment of TI in macrophages.

### 3.5. HBHA Induces Trained Immunity in Human-Derived Innate Immune Cells

To validate the translational relevance of HBHA-induced TI, we extended our investigation to human-derived innate immune cells. THP-1 macrophages and hPBMCs were stimulated with 1 μg/mL or 6 μg/mL of HBHA for 24 h, followed by a 5-day resting period, and subsequently restimulated with LPS for 24 h. The mRNA expression levels of the pro-inflammatory cytokines IL-1β, IL-6, and TNF-α were quantified using qRT-PCR to evaluate the induction of TI.

**Figure 4:**
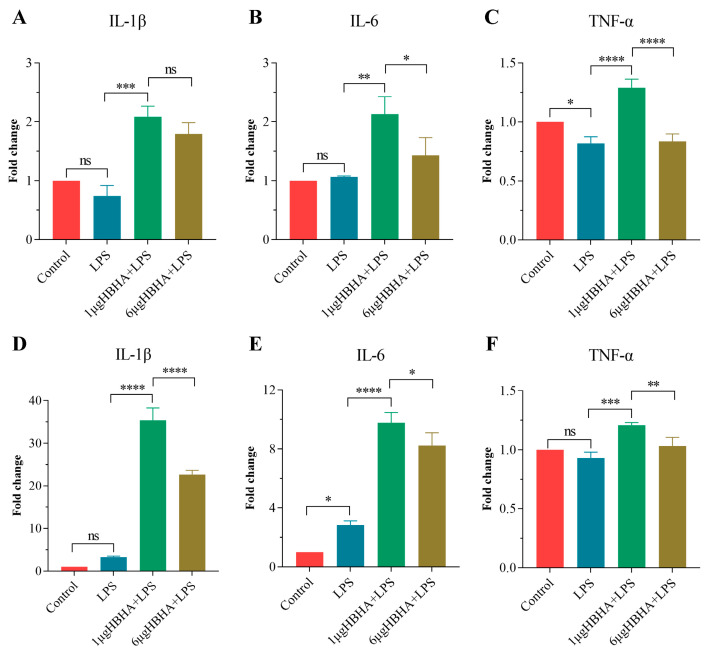
HBHA induces trained immunity in human-derived innate immune cells. (**A**–**C**) Fold change in mRNA expression of IL-1β, IL-6, and TNF-α in THP-1-derived macrophages after training with HBHA (1 or 6 μg/mL) for 24 h, followed by 5-day resting and 24 h of LPS restimulation. The control groups consist of LPS-only treated cells and non-treated control cells. (**D**–**F**) Relative mRNA expression levels of IL-1β, IL-6, and TNF-α in hPBMCs following HBHA training (1 μg/mL or 6 μg/mL) and LPS restimulation. The control groups consist of LPS-only treated cells and non-treated control cells. hPBMCs were isolated from fresh blood of three healthy donors. Data are shown as mean ± SD from three independent experiments (n = 3 biological replicates), each with three technical replicates. One-way ANOVA with Tukey’s post-test was used for multiple comparisons of data in (**A**–**F**). ns, not significant; * *p* < 0.05, ** *p* < 0.01, *** *p* < 0.005, **** *p* < 0.001.

In THP-1 macrophages, training with 1 μg/mL of HBHA significantly amplified the levels of IL-1β, IL-6, and TNF-α mRNA compared to both the LPS-only stimulation and non-treated controls (Figure 4A–C). Notably, higher HBHA concentrations (6 μg/mL) did not further enhance cytokine expression, suggesting a dose-dependent plateau effect in THP-1.

In hPBMCs, training with HBHA robustly upregulated IL-1β and IL-6 expression compared to the LPS-only and non-treated control groups, with a more pronounced effect at 1 μg/mL than at 6 μg/mL (Figure 4D–F). TNF-α expression was also significantly elevated in hPBMCs primed with 1 μg/mL of HBHA, though this effect diminished at 6 μg/mL (Figure 4F).

These findings confirm that HBHA induces trained immunity in human-derived innate immune cells, as characterized by enhanced pro-inflammatory responses upon secondary challenge, highlighting its potential as an effective inducer of macrophage TI.

## 4. Discussion

In this study, we demonstrated for the first time that HBHA, a methylated antigen of Mtb, induces trained immunity in both murine RAW264.7 macrophages and human-derived cells, including THP-1-derived macrophages and hPBMCs. HBHA training significantly enhanced the production of the pro-inflammatory cytokines IL-1β, IL-6, and TNF-α upon secondary stimulation with LPS, accompanied by epigenetic modifications at cytokine gene loci and metabolic reprogramming toward glycolysis. Importantly, HBHA-trained macrophages exhibited the improved control of intracellular mycobacteria, as evidenced by the reduction in CFU following BCG infection, highlighting the functional relevance of TI in antimicrobial defense. These findings establish HBHA as a promising candidate for further investigation in the context of tuberculosis prevention, particularly through mucosal immunization strategies.

The ability of HBHA to induce TI aligns with the established mechanisms observed with other TI inducers, such as BCG and β-glucan, which similarly reprogram innate immune cells through epigenetic and metabolic rewiring [8,36]. In particular, HBHA training increased the deposition of activating histone marks H3K4me1 and H3K4me3 at the promoters/enhancers of IL-1β, IL-6, and TNF-α, facilitating rapid transcriptional responses. Concurrently, metabolic shifts, including elevated lactate production and the upregulation of glycolytic enzymes (e.g., PFKM, PKM, LDHA), support the heightened functional state of trained macrophages. These molecular signatures underscore the robustness of HBHA-induced TI. These molecular changes, which underscore the robustness of HBHA-induced TI, may be mediated through TLR4-dependent signaling cascades, including NF-κB, PI3K–Akt–mTOR, and MAPKs. These pathways are known to drive metabolic rewiring toward glycolysis and the accumulation of metabolites like fumarate and succinate, which in turn inhibit histone demethylases, enriching active histone marks such as H3K4me3 and H3K4me1 at inflammatory gene promoters [4,37]. Future genome-wide epigenomic and metabolic profiling studies will be essential to fully elucidate these pathways.

It is important to note that the effectiveness of HBHA in inducing TI appears to be dose-dependent. In our experiments, a lower concentration of HBHA (1 μg/mL) was more effective than a higher concentration (6 μg/mL) in eliciting certain immune responses, such as cytokine production. This counterintuitive observation may be attributed to potential immune tolerance or metabolic dysregulation at higher concentrations, as suggested by studies on innate immune tolerance where excessive stimulation leads to functional impairment [37,38]. Further investigation is warranted to determine the optimal concentration range for HBHA-induced TI and to elucidate the underlying mechanisms governing this dose-dependent effect.

Unlike live vaccines or complex polysaccharides, HBHA is a purified protein with TLR4 agonist activity, offering a safer and more controllable platform for TI-based interventions. While BCG-induced TI has been extensively studied, HBHA’s role as a TI inducer is underexplored. Our findings not only validate its efficacy but also highlight its potential advantages, such as the reduced risk of adverse effects in immunocompromised individuals. However, it is important to acknowledge potential safety concerns, particularly in individuals with autoimmune or inflammatory diseases, where HBHA-induced trained immunity may exacerbate these conditions. Additionally, given that HBHA may activate innate immune responses, it is crucial to explore its interactions with existing BCG vaccination, as it may either complement or interfere with the vaccine’s efficacy.

Beyond its TI-inducing capacity, HBHA also elicits robust adaptive immune responses, particularly strong T cell responses in latently infected individuals and protective immunity against active tuberculosis, as demonstrated in mouse challenge models [24,25,26]. This dual capacity to induce both innate TI and adaptive memory responses positions HBHA as a unique candidate for TB vaccines. TI in macrophages ensures rapid pathogen containment at the site of infection, while adaptive T cell responses provide long-term, antigen-specific protection. This synergy may offer superior efficacy compared to strategies targeting only one arm of the immune system.

Given that the respiratory mucosa is the primary entry site for Mtb [39], HBHA-induced TI in tissue-resident macrophages could establish early localized immunity. Our validation of the TI effects of HBHA in human-derived cells further supports its translational potential. Future mucosal vaccination strategies incorporating HBHA could leverage both innate and adaptive immune memory to achieve durable protection against TB.

Our in vitro findings demonstrate that HBHA-induced TI confers improved control of intracellular mycobacteria in macrophages, as shown by the significant reduction in CFU post-BCG infection. Although BCG commonly serves as a model for Mtb infection, its attenuation does not fully mimic virulent Mtb. Therefore, future in vivo studies in animal models are required to validate their physiological relevance, particularly in the context of virulent Mtb infection. To address this limitation, we are planning future studies using animal models, such as C57BL/6 mice infected with H37Rv, to assess HBHA’s protective efficacy in a more clinically relevant context. Moreover, while macrophages are central to early TB defense, exploring HBHA’s effects on other innate immune populations like dendritic cells could further illuminate its broader immunomodulatory potential. Additionally, further mechanistic studies exploring the signaling pathways will be crucial to help us better understand the molecular mechanisms driving HBHA’s immunomodulatory effects and refine its therapeutic application. In particular, future studies should include TLR4 inhibition experiments (e.g., pharmacological inhibition or gene silencing) to confirm the involvement of this pathway in HBHA-induced trained immunity, as well as examination of other histone modifications (e.g., H3K27ac, H3K9me3) and measurement of key metabolic intermediates (e.g., fumarate, succinate, lactate) to provide a more comprehensive understanding of the epigenetic and metabolic reprogramming involved in trained immunity.

## 5. Conclusions

In summary, this study demonstrates that HBHA induces trained immunity in macrophages, characterized by coordinated epigenetic and metabolic reprogramming, leading to improved control of intracellular mycobacteria in vitro. These findings suggest that HBHA may serve as a valuable tool for further mechanistic studies of trained immunity and its potential relevance to host defense against mycobacterial infection. Future in vivo experiments and clinical research are necessary to validate and extend these observations, particularly in the context of tuberculosis.

## Figures and Tables

**Table 1:** Sequences for qRT-PCR primers.

Species	Primer Name	Forward Primer (5′ to 3′)	Reverse Primer (5′ to 3′)
Mouse	IL-1β	GCAACTGTTCCTGAACTCAACT	ATCTTTTGGGGTCCGTCAACT
	IL-6	TAGTCCTTCCTACCCCAATTTCC	TTGGTCCTTAGCCACTCCTTC
	TNF-α	CCCTCACACTCAGATCATCTTCT	GCTACGACGTGGGCTACAG
	PFKM	TGTGGTCCGAGTTGGTATCTT	GCACTTCCAATCACTGTGCC
	PKM	GCCGCCTGGACATTGACTC	CCATGAGAGAAATTCAGCCGAG
	LDHA	TGTCTCCAGCAAAGACTACTGT	GACTGTACTTGACAATGTTGGGA
	Cs	GGACAATTTTCCAACCAATCTGC	TCGGTTCATTCCCTCTGCATA
	OGDH	GTTTCTTCAAACGTGGGGTTCT	GCATGATTCCAGGGGTCTCAAA
	IDH1	ATGCAAGGAGATGAAATGACACG	GCATCACGATTCTCTATGCCTAA
Human	IL-1β	ATGATGGCTTATTACAGTGGCAA	GTCGGAGATTCGTAGCTGGA
	IL-6	ACTCACCTCTTCAGAACGAATTG	CCATCTTTGGAAGGTTCAGGTTG
	TNF	GAGGCCAAGCCCTGGTATG	CGGGCCGATTGATCTCAGC

**Table 2:** Sequences for ChIP-qPCR primers.

Antibodies	Target Gene	Forward Primer (5′ to 3′)	Reverse Primer (5′ to 3′)
H3K4me1	IL-1β	ATGAAACCTGTGTGGGAGCC	GCCTTGCTCCCAGGCTATTT
	IL-6	AAGGGCTTCTGGCTACCATTAG	TTGCATCTGGCTTTGTTCGC
	TNF-α	TGCTTGATCTCCCGTTATCTCC	TGTTCACACGTGGAGAGATCTG
H3K4me3	IL-1β	CAACATGGGGAACAGCATTAGG	AGCTCCTGTCTTGTAGGAAAGC
	IL-6	GGGCGTCCATTCATTCTCTTTG	CCACTCAAAACCAGCAAAGAGG
	TNF-α	CCTCATGTCTCTTTGCTCTGC	TTGTGTCTGTCTTGCGTTGG

## Data Availability

The original contributions presented in this study are included in the article/Supplementary Materials. Further inquiries can be directed to the corresponding authors.

## Supplementary Materials

The following supporting information can be downloaded at: https://www.mdpi.com/article/10.3390/biom15070959/s1, Figure S1: Relative mRNA expression of IL-1β, IL-6, and TNF-α in RAW264.7 cells following HBHA stimulation. Figure S2: Comparison of cytokine expression in RAW264.7 macrophages after training with HBHA, BSA, or heat-killed BCG (HKBCG). Figure S3: Expression of pro-inflammatory cytokines at the end of the 5-day rest period after initial HBHA stimulation. Excel S1: Statistical comparisons among all treatment groups in cytokine mRNA expression. File S2: Raw datasets.
