# Supplementary material for: Heparin-Binding Hemagglutinin-Induced Trained Immunity in Macrophages: Implications for Antimycobacterial Defense
Source: Biomolecules. 2025 Jul 4;15(7):959. doi: 10.3390/biom15070959 (PMC12292597; doi:10.3390/biom15070959)
Supplement: Supplementary file 1 [file biomolecules-15-00959-s001.zip › File S1-Supplementary Figure 1-3.pdf]

Supplementary Figures

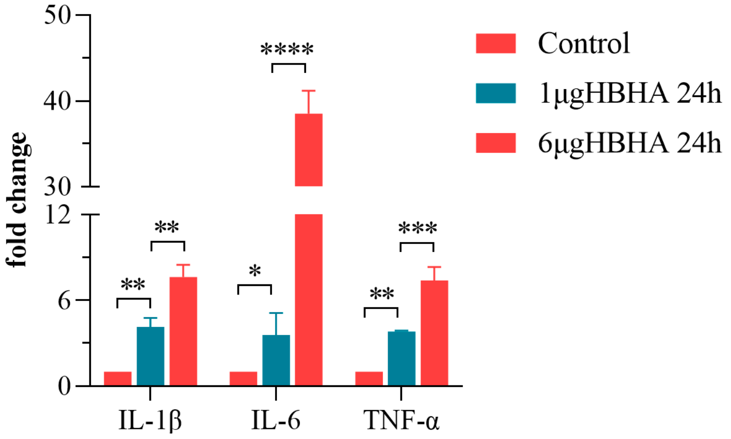

Supplementary Figure S1 Relative mRNA expression of IL-1β, IL-6, and TNF-*α* in RAW264.7 cells following HBHA stimulation. RAW264.7 cells were treated with either 1 μg/mL or 6 μg/mL HBHA for 24 hours, with an unstimulated group serving as the control. mRNA expression levels of IL-1β, IL-6, and TNF-*α* were measured using qPCR. Fold changes in expression were calculated relative to the control group. Data represent mean ± SD from three independent experiments (n=3 biological replicates), each with three technical replicates. Data in (B-G, I) represent mean ± SD from at least three independent experiments (n=3 biological replicates), each with three technical replicates. *p < 0.05, **p < 0.01, ***p < 0.005, ****p < 0.0001.

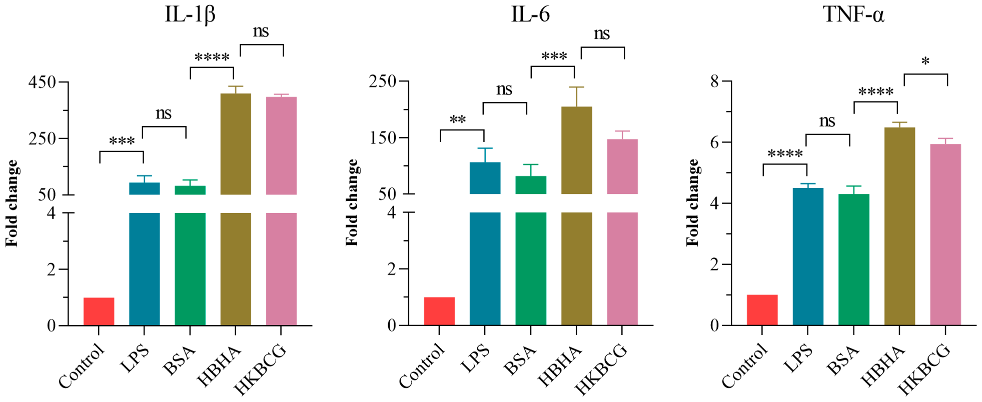

Supplementary Figure S2 Comparison of cytokine expression in RAW264.7 macrophages after training with HBHA, BSA, or heat-killed BCG (HKBCG). RAW264.7 cells were trained with either 1 μg/mL HBHA, 1 μg/mL BSA (negative control protein), or 10 μg/mL heat-killed BCG (HKBCG, positive control) for 24 hours. Control cells received no training. After a 5-day resting period, all groups were restimulated with 10 ng/mL LPS for 4 hours. mRNA expression levels of IL-1β, IL-6, and TNF-*α* were measured by qPCR. Cytokine gene expression is shown as fold change relative to the non-treated control group. Data represent the mean ± SD of three independent biological replicates, each performed with three technical replicates. Statistical analysis was performed using one-way ANOVA followed by Tukey's multiple comparisons

test. *p < 0.05, **p < 0.01, ***p < 0.001, ****p < 0.0001; ns: not significant.

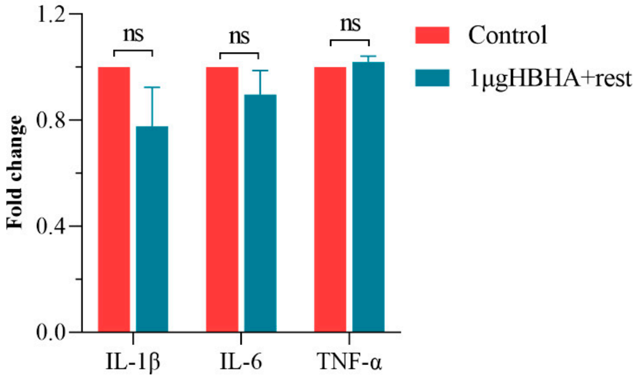

Supplementary Figure S3 Expression of pro-inflammatory cytokines at the end of the 5-day rest period after initial HBHA stimulation. Cells were not re-stimulated. No significant differences were observed in IL-1β, IL-6, or TNF-α mRNA levels between HBHA-treated and control groups, indicating a return to resting state. Data are shown as mean ± SD from three biological replicates. ns = not significant.
