# Supplementary material for: Heparin-Binding Hemagglutinin-Induced Trained Immunity in Macrophages: Implications for Antimycobacterial Defense
Source: Biomolecules. 2025 Jul 4;15(7):959. doi: 10.3390/biom15070959 (PMC12292597; doi:10.3390/biom15070959)

Control			HBHA		
427680	422400	431200	383680	401280	396000
299376	255680	301840	191840	200640	168000
323528	359040	366520	123880	150480	148500

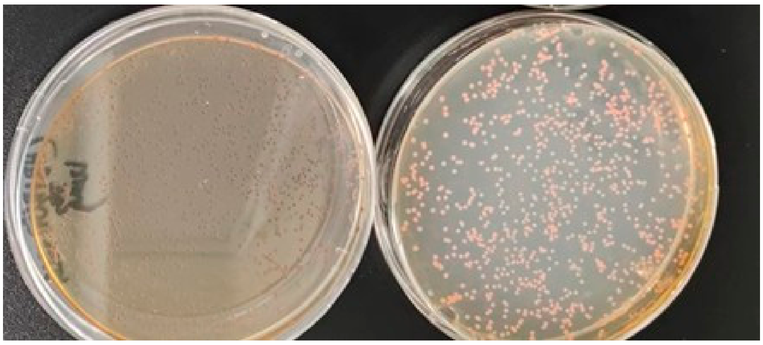

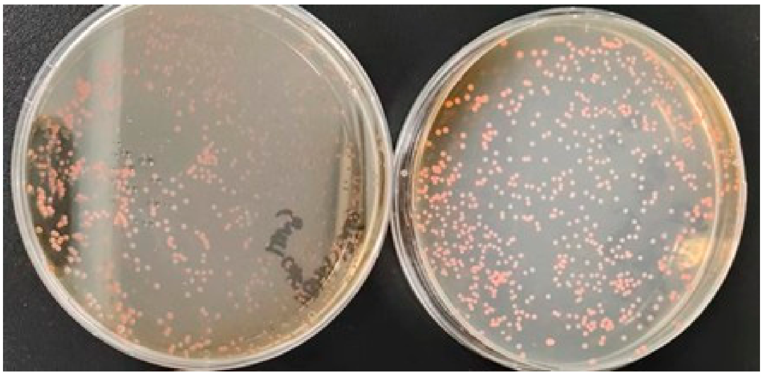

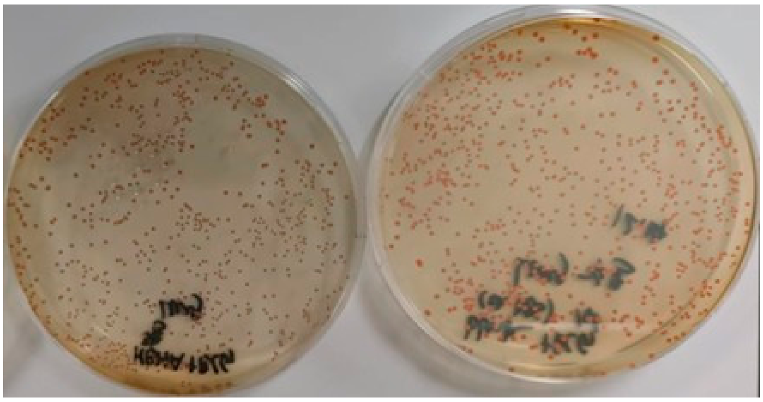

Supplement: Supplementary file 1 [file biomolecules-15-00959-s001.zip › File S2-raw data.zip!/File S2-raw data/raw data/enumeration of CFU/CFU.pdf]
